# Sono-Assembly of the [Arg-Phe]_4_ Octapeptide into Biofunctional Nanoparticles

**DOI:** 10.3390/nano10091772

**Published:** 2020-09-08

**Authors:** Anshul Baral, Sukhvir K. Bhangu, Rita Cimino, Juliane N. B. D. Pelin, Wendel A. Alves, Santanu Chattopadhyay, Muthupandian Ashokkumar, Francesca Cavalieri

**Affiliations:** 1 School of Chemistry, University of Melbourne, Melbourne, VIC 3010, Australia; anshulb@student.unimelb.edu.au; 2 School of Science, RMIT University, Melbourne, VIC 3000, Australia; roop.bhangu@rmit.edu.au; 3 Department of Chemical Sciences and Technologies, University of Rome “Tor Vergata”, 00133 Rome, Italy; Rita.Cimino@uniroma2.it; 4 Centro de Ciências Naturais e Humanas, Universidade Federal do ABC, Santo Andre 09210-580, Brazil; juliane.pelin@ufabc.edu.br (J.N.B.D.P.); wendel.alves@ufabc.edu.br (W.A.A.); 5 Rubber Technology Centre, Indian Institute of Technology, Kharagpur 721302, India; santanu@rtc.iitkgp.ernet.in

**Keywords:** [Arg-Phe]_4_ octapeptides, self-assembly, acoustic cavitation, sono-assembly, nanoparticles

## Abstract

High-frequency ultrasound treatment is found to be a one-pot green technique to produce peptide-based nanostructures by ultrasound assisted self-assembly of oligopeptides. [Arg-Phe]_4_ octapeptides, consisting of alternating arginine (Arg/R) and phenylalanine (Phe/F) sequences, were subjected to 430 kHz ultrasound in aqueous solution in the absence of any external agents, to form [RF]_4_ nanoparticles ([RF]_4_-NPs), ~220 nm in diameter. A comprehensive analysis of the obtained nanoparticles demonstrated that the aromatic moieties of the oligopeptides can undergo oxidative coupling to form multiple oligomeric species, which then self-assemble into well-defined fluorescent nanoparticles. [RF]_4_-NPs were functionalized with polyethylene glycol (PEGylated) to improve their colloidal stability. Unlike the parent peptide, the PEGylated [RF]_4_-NPs showed limited cytotoxicity towards MDA-MB-231 cells. Furthermore, the intracellular trafficking of PEGylated [RF]_4_-NPs was investigated after incubation with MDA-MB-231 cells to demonstrate their efficient endo-lysosomal escape. This work highlights that the combined use of ultrasonic technologies and peptides enables easy fabrication of nanoparticles, with potential application in drug delivery.

## 1. Introduction

Self-assembly of peptides is a fascinating phenomenon that produces a wide range of nanostructures with various functionalities [1,2,3,4]. Peptides can undergo different structural conformations depending on their primary structure to form various secondary structures, such as α-helices, β-sheets, and random coils, which further self-assemble to attain well-defined and stable nanostructures like nanotubes, nanofibers, helical ribbons, and nanoparticles [1,2,3,5]. These nanostructures are stabilized through different non-covalent interactions such as hydrogen bonding, π-π interaction, Van der Waals force, and electrostatic interactions [6]. Peptide-based self-assembled nanostructures have been used in bio-medical applications such bio-diagnostics, gene/drug delivery, and tissue engineering due to their biodegradability and limited toxicity [5,7,8,9,10,11,12,13,14,15,16,17]. Many research efforts have been reported on the controlled synthesis of peptide-based nanostructures using self-assembly under different factors such as pH, metal ion, temperature, and salts [18]. For instance, controlled self-assembly of peptides in the presence of metal ions such as magnesium [18], calcium [19], and potassium [20] has been reported to produce nanoparticles. The importance of amino acid sequence on the structure of peptide-based nanostructures was demonstrated by Hamley and coworkers [21,22]. Notwithstanding all this progress, precise control over the morphologies and functionalities of the peptide-based nanomaterials obtained by spontaneous self-assembly has not been achieved. In addition, the spontaneous self-assembly of peptides often leads to the formation of toxic disordered fibrils [23,24,25].

In this study, we have explored the application of high-frequency ultrasound to promote the controlled self-assembly of peptides into non-toxic nanoparticles without any external agent. Ultrasonics and sonochemistry is an emerging technology which has been successfully applied to the fabrication of a wide range of functional materials [26]. High-frequency ultrasound has proven to be one of the efficient techniques to produce well-defined tunable functional nanoparticles from simple aromatic biomolecules. We recently [27] showed that the cavitation bubble interface acts as a catalytic binding site for coupling reactions among amphiphilic amino acids such as tyrosine, tryptophan, and phenylalanine. To the best of our knowledge, the ultrasound driven self-assembly and crosslinking of peptides to form nanostructures has not yet been reported. Compared to the spontaneous self-assembly of peptides, the ultrasound-mediated self-assembly may offer better control over the stability, shape and size, and cytotoxicity of the nanostructures.

Herein, we report on the effect of high-frequency ultrasound treatment on aqueous solutions of self-assembling oligopeptides. Alves and coworkers [28,29] have extensively explored the spontaneous self-assembly of [RF]_4_ octapeptides comprising arginine (R) and phenylalanine (F) in an alternating sequence. The [RF]_4_ octapeptides can form interconnected nanofibrillar structures rich in β-sheet structures, as well as other oligomeric species by self-assembly via the solid-vapor phase method. We sought to investigate whether the ultrasonic treatment of this peptide may result in the formation of nanoparticles, rather than nanofibrillar structures, via the ultrasound-mediated coupling reactions and π-π interactions of the phenylalanine residues. Stable nanoparticles ([RF]_4_-NPs) using high-frequency ultrasound treatment of 430 kHz were obtained. A comprehensive characterization of the sono-assembled [RF]_4_-NPs was performed to propose a possible mechanism for the formation of nanoparticles. The observed results showed that [RF]_4_ octapeptides underwent coupling reactions to form multiple oligomeric species under high-frequency ultrasound treatment, and, subsequently, the oligomeric species self-assembled to form nanoparticles. Furthermore, in-vitro studies were performed to verify the non-toxicity and endosomal escape of [RF]_4_-NPs towards cytosols due to the proton sponge effect. The observed in-vitro cytotoxicity and intracellular trafficking results suggest that the synthesized [RF]_4_-NPs can be potentially used as a nanocarrier for controlled drug delivery.

## 2. Materials and Methods

[RF]_4_ octapeptides (Figure S1) were synthesized in our lab [28]. We procured 1-diphenyl-2-picrylhydrazyl (DPPH), Dulbecco’s phosphate-buffered saline (D-PBS), bovine serum albumin (BSA), hydrogen peroxide (30%), and EIPA (ethylisopropyl amiloride) from Sigma Aldrich (St. Louis, MO, USA). Methanol (99.9%) and HPLC grade formic acid were bought from Fisher chemicals (Hampton, NH, USA). Phalloidin Alexa 480, rabbit anti-EEA-1 monoclonal antibody, and rabbit anti-Rab7 monoclonal antibodies were purchased from Cell Signalling Technology (Beverly, MA, USA). 3-(4,5-dimethylthiazol-2-yl)-2,5-diphenyl tetrazolium bromide (MTT) reagent, Rabbit LAMP-1 and goat anti-rabbit IgG secondary antibody Alexa Fluor 647 were provided by Invitrogen (Carlsbad, CA, USA). Fetal bovine serum (FBS) was procured from Bovogen (Victoria, Australia). Dulbecco’s Modified Eagle’s medium (DMEM) was obtained from Lonza (Basel, Switzerland). PEG 1000, DMSO-d_6_, and HPLC grade ACN were purchased from MERCK chemicals (Darmstadt, Germany). All solutions were prepared in Milli-Q water with a resistivity of 18.2 MΩ/cm, unless otherwise stated.

[RF]_4_ octapeptides were dissolved in Milli-Q water at different concentrations to determine their critical aggregation concentrations (*cac*) for selecting the suitable solution concentration to carry out further experimentation. Critical aggregation concentrations (*cac*) was determined using the pendant drop technique on OCA 15 EC (DataPhysics Instruments GmbH, Filderstadt, Germany) in a water/air system by measuring the interfacial tension (IFT) at different concentrations of [RF]_4_ solutions. Computer automation allows rapid drop image acquisition, edge detection, and fitting by Young-Laplace equation to determine the interfacial tension. A concentration of 1 mg/mL of [RF]_4_ octapeptides was judiciously selected from the *cac* results. The sonication setup consisted of a double-walled glass cell sonication bath mounted on an ELAC Nautik USW 51-052 transducer of diameter 5.4 cm powered by T&C Power Conversion, Inc. (Rochester, NY, USA). The samples were sonicated in a sonication bath of 200 mL water using 430 kHz frequency and 60 W acoustic power at a constant temperature of 37 ± 2 °C. The reactions were studied as a function of sonication time.

A comprehensive analysis of sonicated samples was performed using different characterization techniques like fluorescence spectroscopy, high-performance liquid chromatography (HPLC), fluorescence microscopy, size exclusion chromatography (SEC), mass spectroscopy (MS), scanning electron microscopy (SEM), and ^1^H nuclear magnetic resonance (NMR). A Shimadzu RF-5301PC fluorescence spectrophotometer was used to acquire fluorescence spectra, using a slit width of 5 nm for excitation and 5 nm for emission spectra. An Agilent Infinity 1260 high-performance liquid chromatography (HPLC) (Agilent Technologies, Santa Clara, CA, USA) unit equipped with a Phenomenex column, model “Jupiter 5u C18 300A” (Phenomenex, Torrance, CA, USA), was used to perform HPLC analysis for the native and sonicated [RF]_4_ octapeptide solutions. The eluent consisted of Milli-Q water (100%) and acetonitrile HPLC grade (100%), denoted as solvent A and solvent B, respectively. The following parameters were used for HPLC analysis: injection volume: 20 μL; flowrate: 1 mL/min; gradient: 0% to 100% of solvent B in 20 min, and UV detector emitting at 260 nm. Size exclusion chromatography (SEC) was performed using a TSKgel G3000SWXL column no. 07S04249C (MERCK & Co., Kenilworth, NJ, USA) for native and sonicated [RF]_4_ solutions, respectively. A mixture of 0.1 mol/L Na_2_SO_4_ with 0.05% NaN_3_ in 0.1 mol/L phosphate buffer of pH 6.7 was used as the eluent for SEC study. The morphology of the particles was inspected using the FEI Teneo VolumeScope Scanning Electron Microscope (Thermo Fisher Scientific, Waltham, MA, USA). Samples were prepared by sputter-coating with a thin layer of gold. ^1^H NMR spectra of [RF]_4_ and [RF]_4_-NPs were obtained in DMSO-d_6_ solvent using Varian MR400 NMR spectrometer (Agilent Technologies) at 400 MHz at 25 °C. A Perkin Elmer AxION^®^ 2 ToF quadrupole mass spectrometer (Perkin Elmer, Waltham, MA, USA) was used for carrying out mass analysis of the sonicated [RF]_4_ solutions. Gradient elution was carried out with solvent A (0.1% formic acid) and solvent B (acetonitrile with 0.1% formic acid) at 30 °C. The zeta potential and hydrodynamic diameter of nanoparticles were determined using ZEN0040, Malvern Instruments (Malvern, UK). Stability of the [RF]_4_-NPs was measured using 100 µL (1 mg/mL) solution of nanoparticles suspended in PBS solution (pH = 7.4) by comparing the hydrodynamic diameter of particles over a period of 4 days.

The cytotoxicity of the nanoparticles was estimated using MTT assay. MDA-MB-231 cells (ATCC^®^ HTB-26^TM^, Manassas, VA, USA) were plated on 96-well plates (Costar 3596, Corning, MA, USA) with a seeding density of 10,000 cells per well in 100 μL of DMEM medium supplemented with 10% fetal bovine serum (FBS). Cells were incubated at 37 °C for 24 h. Then, 1 mg/mL stock solution of [RF]_4_ octapeptides and PEGylated [RF]_4_-NPs was prepared and diluted in accordance with the MTT cell viability assay protocol. The final concentrations were: 0.5, 0.2, 0.1, 0.05, 0.002, 0.0125, 0.006, 0.003, and 0.0015 mg/mL. Afterwards, [RF]_4_ octapeptides and PEGylated [RF]_4_-NPs were added to the culture media and incubated for 24 h. Cell viability was determined by measuring the absorbance at 554 and 670 nm as a reference by Infinite M200 microplate reader (Tecan, Switzerland). For the intercellular trafficking study, MDA-MB-231 cells were seeded at seeding density of 40,000 cells per well in Nunc^TM^ Labtek 8-well chamber coverglass slides (ThermoFisher Scientific, Scoresby, Australia) and incubated overnight at 37 °C and 5% CO_2_. The medium was replaced, and PEGylated RF_4_-NPs were added to a final concentration of 0.075 mg/mL. The nanoparticles were incubated for 5 h, and medium was replaced and further incubated for 9, 12, and 24 h. After the desired incubation time, the medium was removed, and cells were washed three times with PBS-BSA (1% BSA in PBS) to remove unbound particles. Cells were fixed with 4% paraformaldehyde for 15 min at room temperature, washed, permeabilized with 0.1% TritonX-100 solution in PBS for 5 min, and washed three times with PBS. The samples were blocked with 2.5% PBS-BSA for 1 h. Afterwards, samples were incubated for 2 h with different primary antibodies, namely rabbit anti-EEA1 monoclonal antibody, rabbit anti-Rab7 monoclonal antibody for the early and late endosome (2 μg/mL), respectively, and mouse anti-LAMP-1 for lysosome (1 μg/mL). After 2 h, cells were washed thrice and incubated for 1.5 h with goat anti-mouse (for lysosome) or goat anti-rabbit (for early and late endosome) IgG secondary antibody Alexa Fluor 647 antibody (2 μg/mL). Cells were then imaged using Nikon A1R confocal microscope with a 60 × 1.4NA oil immersion objective. Dissolution study was performed using 75 µg/mL of [RF]_4_-NPs in PBS solution of pH 5 and pH 7.4 separately at 37 °C. The [RF]_4_-NPs were centrifuged out, and the fluorescent peaks at 435 nm were measured for the supernatant, followed by mixing the centrifuged [RF]_4_-NPs into the supernatant solution. The following procedure was repeated at different intervals of time for a period of 24 h.

## 3. Results and Discussion

**Figure 1:**
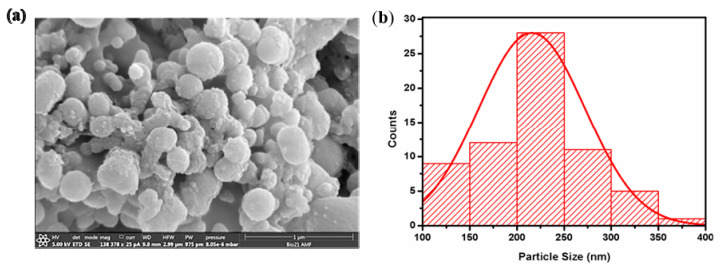
(**a**) SEM image and (**b**) particle size distribution curve for the RF_4_-NPs.

The alternating arginine-phenylalanine [RF]_4_ octapeptide tends to self-assemble and forms *β*-sheet amyloidal fibrils with different amyloidal analogues [28] at a concentration of 0.17 wt.%. The transmittance peaks at 1670 cm^−1^ and 1633 cm^−1^ shown in Figure S2 indicate the *β*-sheet secondary structural conformation of the octapeptide. The other characteristic Fourier Transform Infrared Spectroscopy (FTIR) bands of the [RF]_4_ octapeptides are listed in Table S1. As [RF]_4_ octapeptides can spontaneously self-assemble to form aggregates, the critical aggregation concentrations (*cac*) was first measured. The *cac* of the peptides in millipore water was found to be 1 mM (1.26 mg/mL or 0.126 wt.%) from the plot shown in Figure 1a. [28]. When dissolved in water at a concentration of 0.4 mM (below *cac*), the [RF]_4_ octapeptide exhibited emission peaks at 435, 565, and 620 nm, as shown in Figure 1b. As phenylalanine does not exhibit any fluorescence in the wavelength range of 400–700 nm, the emission peak at 435 nm was attributed to intramolecular π-π interactions between the phenylalanine moieties, whereas the emissions at wavelengths >565 nm might be due to cation-π interactions between the NH^+^ group of arginine and the phenyl ring of the phenylalanine [28]. We performed the sonication of the [RF]_4_ octapeptide at a concentration of 1 mg/mL, below the *cac*, in order to rule out any possibility of aggregation before sonication.

**Figure 2:**
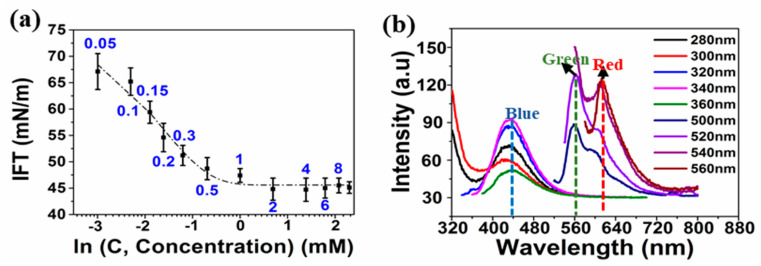
Plot of (**a**) interfacial tension (IFT) as a function of concentration, (**b**) fluorescence emission spectra at different excited wavelengths (very weak emission peaks were obtained in the emission range 520–720 nm as compared to intensity of peak observed at 435 nm; therefore, emission slit width was increased from 5 to 10 nm).

[RF]_4_ octapeptide solutions were subjected to high-frequency ultrasound treatment of 430 kHz for 6 h. The obtained [RF]_4_-NPs were centrifuged, thoroughly washed, and analyzed by SEM (Figure 2a). The [RF]_4_-NPs size distribution was measured (Figure 2b) from SEM images. The average diameter and ξ potential of the nanoparticles were found to be 216 ± 57 nm and +39 ± 6 mV, respectively, in Milli-Q water (pH around 6). The nanoparticles were positively charged due to the protonation of NH_2_ groups below the isoelectric point (pI = 8.93), as shown in the titration curve (Figure S3). The hydrodynamic diameter of the [RF]_4_-NPs (Table S2) was found to be consistent over a period of 4 days, which indicates the stability of the synthesized nanoparticles.

**Figure 3:**
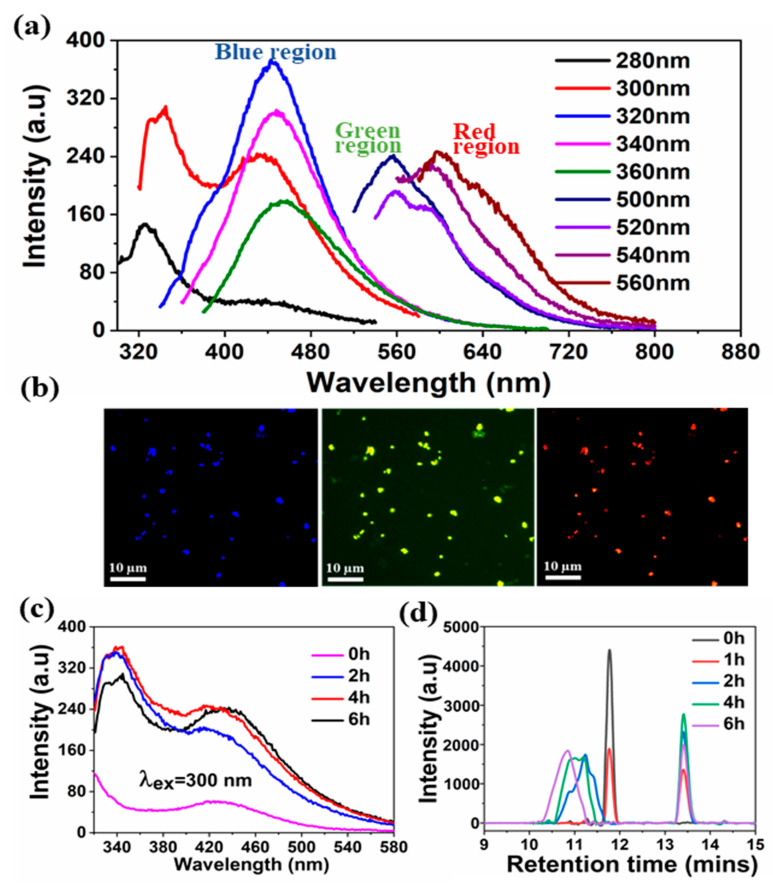
(**a**) The dependence of fluorescence emission spectra on the excitation wavelengths from 300 to 560 nm of the [RF]_4_-NPs, (**b**) the fluorescence microscopic images of [RF]_4_-NPs showing the blue, green, and red fluorescence, (**c**) the fluorescence emission of the [RF]_4_-NPs at λ_ex_ = 300 nm at different sonication times, and (**d**) HPLC plot for the sonicated products at different sonication times.

**Figure 4:**
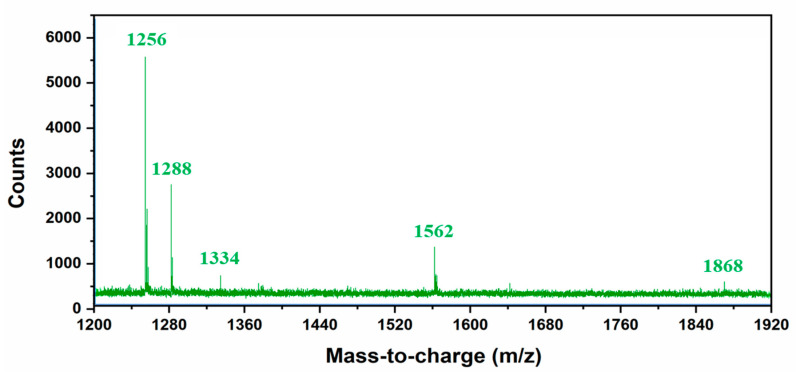
Mass spectroscopy data of the sonicated RF_4_ peptide in higher molecular weight range.

**Figure 5:**
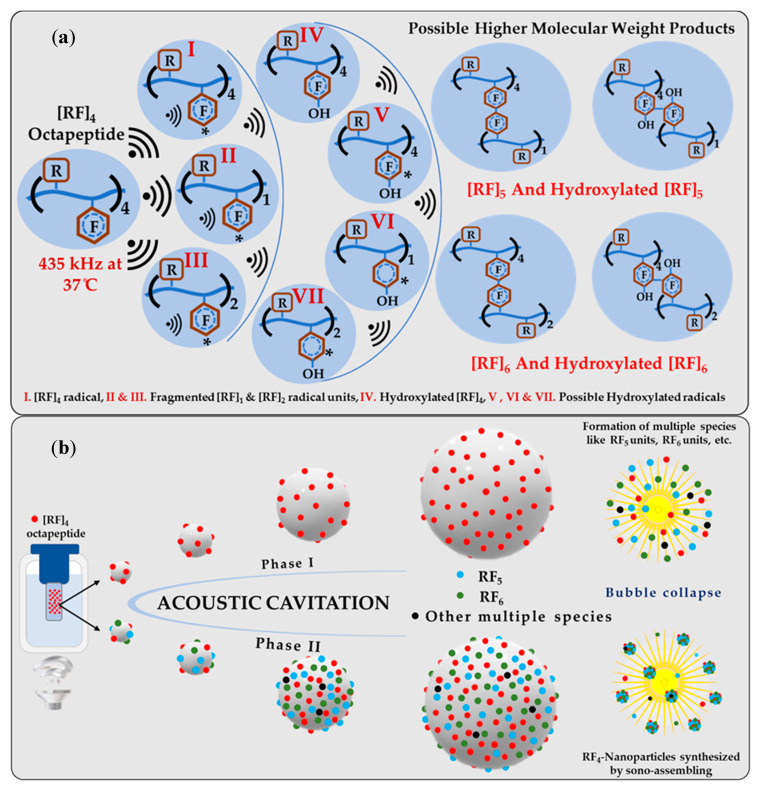
Schematic representation of (**a**) the possible chemical reactions carried out during the high-frequency ultrasound treatment of [RF]_4_ octapeptides and (**b**) the possible mechanism involved, starting from the sonication of the [RF]_4_ octapeptide molecule to form different oligomeric species (phase I) which act as the fuel for phase II to form sono-assembled [RF]_4_-NPs.

A comprehensive analysis was performed to provide insight into the mechanism involved in the formation of sono-assembled [RF]_4_-NPs. Similar to native peptide, the sono-assembled [RF]_4_-NPs exhibited fluorescence emission in the blue, green, and red regions when excited at different wavelengths (Figure 3a), which was further confirmed by fluorescence microscopic images, as depicted in Figure 3b. High-frequency ultrasound treatment lead to the formation of radicals, which further generate oligomeric species with new functional properties [30,31]. The absorption spectra of the sonicated [RF]_4_ solution, acquired as a function of sonication time (Figure S4), shows a shift in the absorption peak from 260 to approximately 285 nm, along with additional bands appearing between 300 and 400 nm. The intensity of the peaks increased as a function of sonication time. These peaks could be attributed to the π-π* electronic transitions in the compounds with aromatic moieties, where new absorption bands at higher wavelengths may be due to enhanced conjugation of the aromatic moieties [32,33]. In fluorescence spectroscopic analysis, a new emission peak was identified at 340 nm for the [RF]_4_-NPs shown in Figure 3c after sonication, which can be ascribed to the formation of dimers or the hydroxylation of the parent molecule or both [30]. The emission peak intensifies and shifts towards the higher wavelength region with an increase in sonication time, as shown in Figure 3c, providing evidence for multiple species formed with sonication. The improved emission intensities for the sonicated products at wavelengths >560 nm could be attributed to the enhanced intermolecular interactions among the modified peptide molecules upon formation of nanoparticles due to self-assembly during sonication. On the other hand, unsonicated [RF]_4_ octapeptides had a negligible shift in the emission wavelengths at 435, 565, and 620 nm for the different excited wavelengths, as shown in Figure 1b; however, there were significant shifts in the emission peaks observed for the sonicated products, as shown in Figure 3a. This dependence of the emission spectrum on the exciting wavelength could be due to the formation of hydroxylated compounds, dimers, trimers, or other high molecular weight species formed upon sonication of the [RF]_4_ octapeptides. The interface of the cavitation bubbles generated by high-frequency ultrasound in the range of 0.35–1 MHz can act as the catalytic binding site for the radical-mediated C-C coupling of phenolic moieties, resulting in the formation of higher molecular weight species [27,30]. To further confirm the formation of high molecular weight species, HPLC, SEC, and mass spectrometry analysis were performed. Figure 3d shows the HPLC profile of [RF]_4_ octapeptides at different sonication times. The appearance of a new elution peak at a higher retention time of 13.5 min confirms the formation of a hydrophobic species with an increase in sonication time. This peak increased as a function of sonication time. The hydrophobic species can be ascribed to the higher molecular weight species produced by sonication of the parent [RF]_4_ octapeptides. The parent [RF]_4_ octapeptides’ retention peak at 11.8 min decreased with sonication time and shifted to the hydrophilic region, which could be due to the hydroxylation and/or degradation of [RF]_4_ octapeptide molecules. The size exclusion chromatography results (Figure S5) also confirm the formation of higher molecular weight species (4.5 min). Mass spectroscopy analysis was performed to gain further insight into the formation of different high molecular weight species upon sonication of [RF]_4_ octapeptide molecules. Table 1 summarizes the different possible high molecular weight products deduced from the MS results (Figure 4). Fragmentation patterns in the lower m/z region owing to the complexity of the sample were observed. Overall, a clear indication of hydroxylation of the parent peptide accompanied with different high molecular weight species formed during the sonication of [RF]_4_ octapeptides was observed. The MS results showed that the final products were formed by the coupling of the parent [RF]_4_ octapeptides with [RF] and [RF]_2_ fragments to form higher molecular weight species, as shown in Figure 5a. 

Further investigations were performed using ^1^H NMR spectroscopy to investigate the possible reactions which occurred during the sonication of [RF]_4_ octapeptides. Figure S6 shows the whole range of ^1^H NMR chemical shifts for the parent and sonicated [RF]_4_ octapeptides dissolved in DMSO-d_6_ solvent. The appearance of chemical shifts at 10.18 ppm after sonication provides a clear indication of hydroxylation in the aromatic side chain of the [RF]_4_ octapeptides. There were significant changes observed in the aromatic region with the sonication of [RF]_4_ octapeptides, as shown in Figure S7. These changes can be ascribed to the formation of hydroxylated or crosslinked phenylalanine residues. These findings support the hypothesis that cavitation bubbles generated by high-frequency ultrasound can act as catalytic binding sites for the radical-mediated C-C coupling of the aromatic moieties [27,30,31].

A possible mechanism for the formation of sono-assembled [RF]_4_-NPs was proposed. Ultrasound under specific experimental conditions leads to acoustic cavitation, in which bubbles grow and reach a resonance size range, where they may either collapse violently within a single pulsating cycle or collapse after oscillating at its resonance size for many cycles, generating a very high localized temperature (above 5000 K) and pressure (above 1000 atm) [34,35]. Under these intense conditions, highly reactive radicals are generated which initiate chemical reactions and processes. Figure 5a represents the overall schematics for the possible chemical reactions carried out during the sonication process. During sonication, OH/H radicals generated abstract the hydrogen atom from the aromatic side chain of the [RF]_4_ octapeptides to form the initiating [RF]_4_ radical, leading to further reactions. As supported by experimental results, OH radicals combined with the [RF]_4_* radicals form hydroxylated-[RF]_4_ molecules. In addition, the ultrasound treatment induced the formation of fragments that can further recombine with [RF]_4_ via C-C coupling to form [RF]_5_ and [RF]_6_. A schematic of the process is shown in Figure 5a. These species ultimately self-assemble into nanoparticles during the collapse of the cavitation bubbles due to the strong π–π interaction by aromatic group in the phenylalanine and cation–π interactions of NH^+^ group present in the arginine with the phenyl ring of the phenylalanine. It is worth noting that a further multi-micellar aggregation (MMA) [36,37] of thermodynamically unstable nanoaggregates of [RF]_5_ and [RF]_6_ into larger nanoparticles is also possible after sonication. 

**Figure 6:**
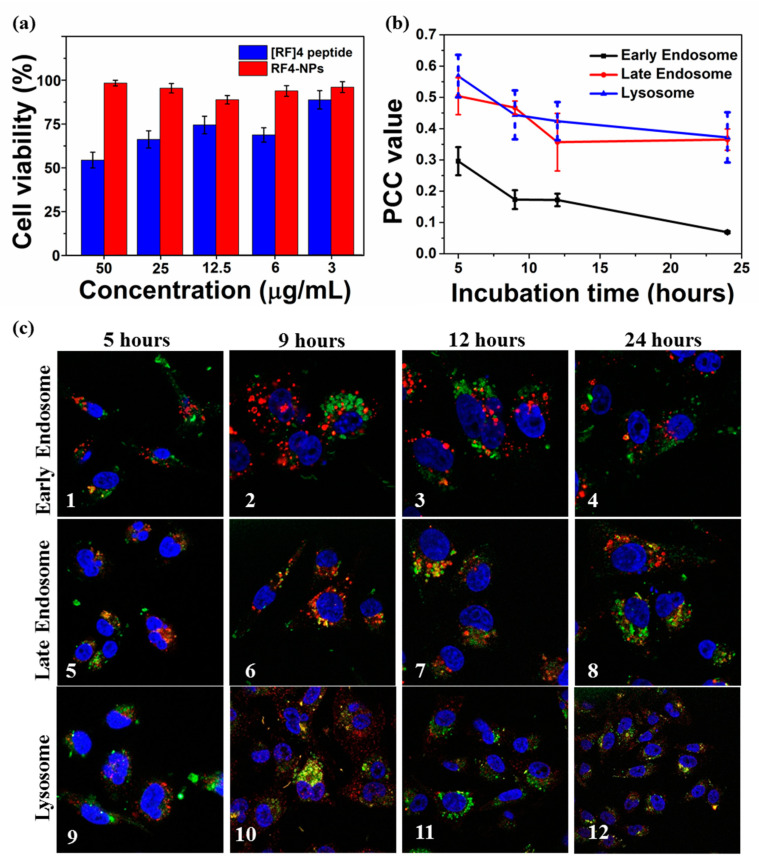
(**a**) Cell viability assay performed on MDA-MB-231 cell line after 24 h incubation with PG-LA 10% using the [RF]_4_ peptide solution and [RF]_4_-NPs, (**b**) PCC values of PEGylated [RF]_4_-NPs in early endosome, late endosome, and lysosome as a function of incubation time, and (**c**) Colocalization studies of PEGylated [RF]_4_-NPs (green) with different intracellular vesicles (red) and cell nuclei (blue) after 5, 9, 12, and 24 h of incubation time.

PEGylation of nanoparticles can enhance the delivery efficiency, prolonged circulation time, and improved stability in biomedicines [38]; therefore, [RF]_4_-NPs were PEGylated to further improve the functionality of the nanoparticles. After PEGylation, the nanoparticles were found to be more dispersed, as shown in Figure S8a, and the particle size reduced to the range of 160–170 nm, as depicted in Figure S8b. This suggests that PEG prevented the aggregation of [RF]_4_-NPs nanoparticles formed after sonication. The z-average particle was measured to be 198 ± 5 nm, and the ξ potential of the nanoparticles was reduced to + 26 ± 6 mV due to the steric shielding effect of PEG. To test the cytotoxicity of [RF]_4_-NPs, a cell viability study was carried out on the MDA-MB-231 cell line after 24 h of incubation at different concentrations of PEGylated [RF]_4_-NPs. It was found that the PEGylated [RF]_4_-NPs did not exhibit any toxicity, even at the concentration of 50 µg/mL; on the contrary, the parent peptide killed 50% cells at the same concentration. Flexible free [RF]_4_ octapeptide chains could form a layer over the cells, disrupting the transmembrane activities [39]; however, [RF]_4_ octapeptides were transformed from flexible polymeric chains into nanoparticles, which could explain the improvement of cell viability for the PEGylated [RF]_4_-NPs. To understand the intercellular trafficking pathway of the particles, PEGylated [RF]_4_-NPs were incubated with MDA-MB-231 cells for 5 h, washed, and further incubated until 9, 12, and 24 h had lapsed. Immunostaining of early, late endosome, and lysosome was carried out using EEA 1, Rab 7, and LAMP-1 markers, respectively. Figure 6c shows the illustrative confocal microscopy imaging of the intrinsically fluorescent PEGylated [RF]_4_-NPs (green signal), cell nuclei (blue signal), and respective cell vesicles (red signal). The Pearson correlation coefficient (PCC) values were calculated to determine the degree of colocalization between cellular vesicles and [RF]_4_-NPs at different incubation times (Figure 6c). The PCC values and corresponding confocal microscopic images (Figure 6b–1–4) indicate negligible colocalization with early endosomes at all incubation times. The respective PCC values for Figure 6c–5–6 (0.50–0.477) and Figure 6c–9–10 (0.57–0.44) indicates the partial colocalization of [RF]_4_-NPs with late endosomes and lysosomes after 5 and 9 h of incubation time, which decreased (up to 0.35) with further incubation times (12–24 h). The PCC values decreased progressively with incubation time for all the organelles, suggesting that the endosomal escape of [RF]_4_-NP towards the cytosol progressively occurs between 5 and 9 h of incubation. The observed endosomal escape can be attributed to the proton sponge effect which comes into effect for histidine-like molecules with pK_a_ of around 6 and other biomolecules exhibiting buffering capacity between pH 5 and 7 [40,41]. It was observed from the potentiometric titration (Figure S3) that peptide exhibits both the buffering capacity between pH 5 and 7 and pK_a_ around 6.4 (ascribed to the hydroxyl groups in [RF]_4_-NPs), which is the endosomal “pH change window”. In addition, [RF]_4_-NP peptide can also escape due to the dissolution at pH 5 within the endo-lysosomal compartments (Figure S9). These factors would create an osmotic imbalance within the endosome and ultimately lead to the disruption of the endosomal membrane to release the payload in the cytosol. Alternatively, the dissolved peptide can diffuse through the membrane to escape the endosome. After escaping to cytosol, the [RF]_4_-NPs can also undergo dissolution under physiological conditions over prolonged periods (Figure S9). From the dissolution kinetics, studied in a test tube in PBS 7.4, ~50% (38 µg/mL), [RF]_4_-NPs were dissembled in 24 h, as shown in Figure S9. Overall, owing to the, stability, fluorescence properties, nanometer size, negative surface charge, and non-toxicity, [RF]_4_-NPs can be a suitable tool for loading and sustained delivery of hydrophobic or negatively charged drugs, such as chemotherapeutics of nucleic acids.

## 4. Conclusions

We have demonstrated that the high-frequency ultrasound treatment can trigger the transformation of self-assembling [RF]_4_ octapeptides into well-defined nanoparticles (RF_4_-NPs). The collapse of acoustic cavitation bubbles prompts the hydroxylation and coupling of [RF]_4_ octapeptides to produce hydroxylated species and high molecular weight species, which gradually self-assembled to form [RF]_4_-NPs. These [RF]_4_-NPs were PEGylated to improve the stability and functionality of these nanoparticles for in-vitro studies. [RF]_4_-NPs have intrinsic fluorescence properties which can be utilized in bio-imaging applications. The in-vitro cytotoxicity and intracellular trafficking results suggest that the synthesized [RF]_4_-NPs can be a potential nanocarrier for drug delivery applications. In addition, this work could introduce the idea of using high-frequency ultrasound as an alternative green technique to fine-tune the nanostructures produced from self-assembling oligopeptides, without the usage of any external agents like metal ion complexes and salts.

## Figures and Tables

**Table 1:** Different possible high molecular weight products deduced from MS data.

m/z	Possible High Molecular Weight Fragments
1256	[RF]_4_ peptide + 2 OH
1288	[RF]_4_ peptide + 4 OH
1334	Fragmented unit of the hydroxylated dimer with charge 2.
1562	[RF]_4_ peptide + [RF] fragmented unit + 2 OH
1868	[RF]_4_ peptide + [RF]_2_ fragmented unit + 2 OH

## Supplementary Materials

The following are available online at https://www.mdpi.com/2079-4991/10/9/1772/s1, Figure S1: Chemical structure of a [RF]_4_ octapeptide, Figure S2: FTIR spectrum of the [RF]_4_ octapeptide, Figure S3: Titration curve of the [RF]_4_-NPs after dissolution showing changes in pH in function of OH-content in aqueous solution, Figure S4: The absorption spectra of the [RF]_4_ solution, acquired as a function of sonication time, Figure S5: SEC curve of the sonicated [RF]_4_ octapeptide as a function of sonication time, Figure S6: ^1^H NMR plot of (a) [RF]_4_ peptide, and (b) sonicated [RF]_4_ peptide, Figure S7: ^1^H NMR chemical shifts in the aromatic region for (a) [RF]_4_ octapeptide, and (b) [RF]_4_-NPs, Figure S8: (a) SEM image, and (b) particle size distribution curve for the PEGylated [RF]_4_-NPs, Figure S9: Dissolution kinetics of [RF]_4_-NPs at 37 °C at different pH values as a function of time suggesting slow dissolution of [RF]_4_-NPs at pH 5.0 and pH 7.4, Table S1: Characteristic FTIR bands of the [RF]_4_-octapeptide, Table S2: Hydrodynamic diameter of [RF]_4_-NPs in PBS solution over time.
