# Supplementary material for: Sono-Assembly of the [Arg-Phe]4 Octapeptide into Biofunctional Nanoparticles
Source: Nanomaterials (Basel). 2020 Sep 8;10(9):1772. doi: 10.3390/nano10091772 (PMC7558974; doi:10.3390/nano10091772)
Supplement: Supplementary file 1 [file nanomaterials-10-01772-s001.pdf]

Supplementary Information

Sono-Assembly of the [Arg-Phe]4 Octapeptide into Biofunctional Nanoparticles

Anshul Baral [1], Sukhvir K. Bhangu [2], Rita Cimino [3], Juliane N. B. D. Pelin [4], Wendel A. Alves [4], Santanu Chattopadhyay [5], Muthupandian Ashokkumar [1,*], and Francesca Cavalieri [2,3,*]

[1] School of Chemistry, University of Melbourne, VIC 3010, Australia; anshulb@student.unimelb.edu.au
[2] School of Science, RMIT University, Melbourne, VIC 3000, Australia; roop.bhangu@rmit.edu.au
[3] Department of Chemical Sciences and Technologies, University of Rome "Tor Vergata", Rome 00133, Italy; Rita.Cimino@uniroma2.it
[4] Centro de Ciências Naturais e Humanas, Universidade Federal do ABC, Santo Andre 09210-580, Brazil; juliane.pelin@ufabc.edu.br (J.N.B.D.P.); wendel.alves@ufabc.edu.br (W.A.A.)
[5] Rubber Technology Centre, Indian Institute of Technology, Kharagpur 721302, India; santanu@rtc.iitkgp.ernet.in
* Correspondence: masho@unimelb.edu.au (M.A.); francesca.cavalieri@rmit.edu.au (F.C.)

Received: 13 August 2020; Accepted: 01 September 2020; Published: 08 September 2020

1. Molecular Structure of [RF]4 Octapeptide

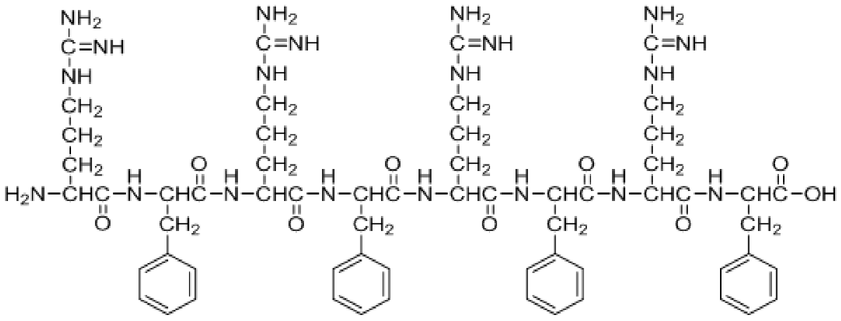

Figure S1. Chemical structure of a [RF]4 octapeptide.

2. FTIR Analysis of the [RF]4 Octapeptides

Table S1. Characteristic FTIR bands of the [RF]4-octapeptide [1].

Approximate frequency (cm^{-1})	Description
3410, 3291	Amide A; NH stretching
2941	Amide B; NH stretching
1670 & 1633	Amide I; C=O stretching, Characteristic peak for β-sheet secondary structure
1480-1575	Amide II; CN stretching, NH bending, 1439; aromatic ring vibration.
1204, 1384	Amide III; CN stretching, NH bending
1134	CN stretching
840	COO^- rocking
698	C-H bending of aromatic ring

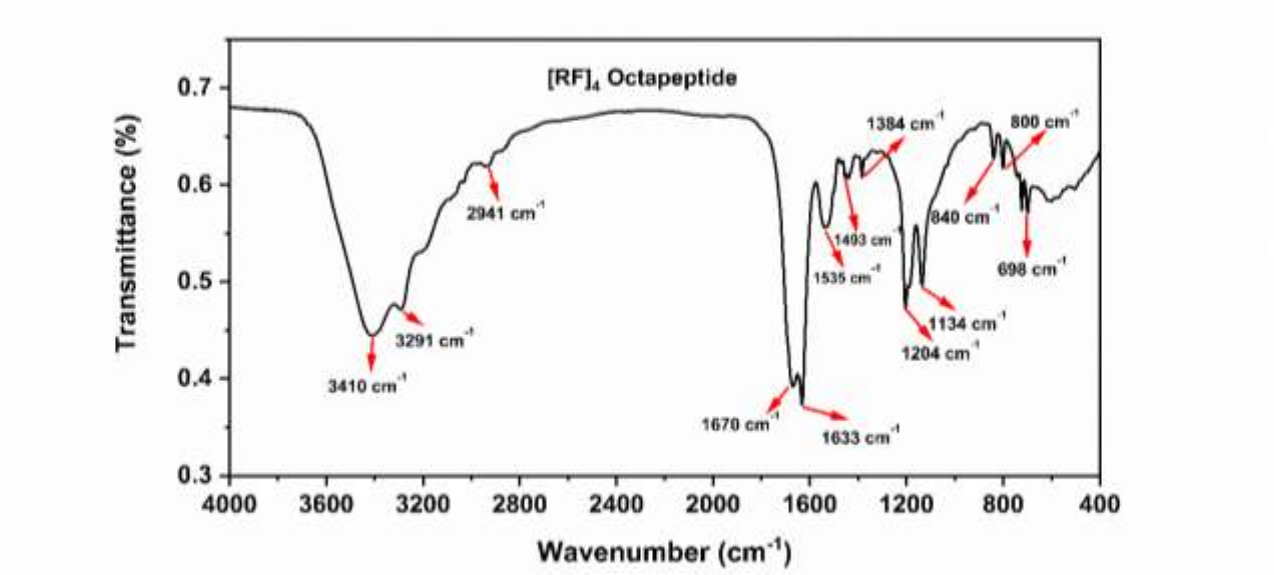

Figure S2. FTIR spectrum of the $[RF]_4$ octapeptide.

3. Potentiometric Titration

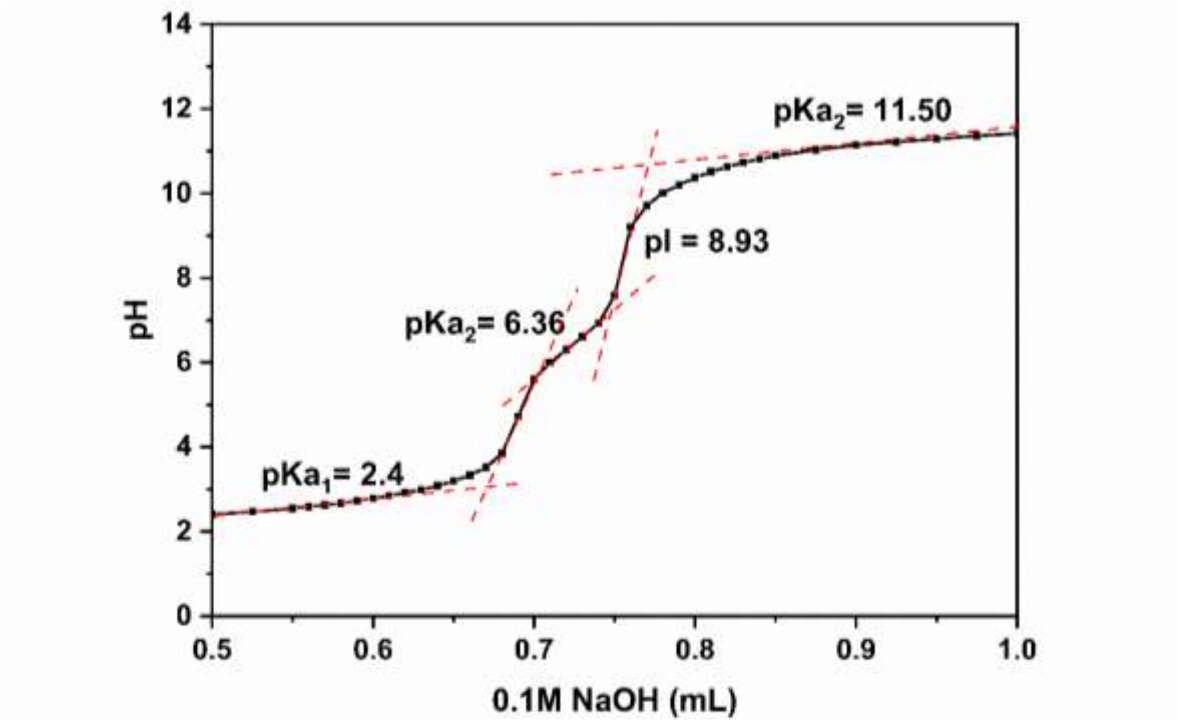

Figure S3. Titration curve of the $[RF]_4$-NPs after dissolution showing changes in pH in function of OH^- content in aqueous solution.

4. Dynamic Light Scattering Analysis

Table S2. Hydrodynamic diameter of $[RF]_4$-NPs in PBS solution over time.

Measurement Day	1	2	3	4
Hydrodynamic Diameter	250 ± 8	256 ± 3	254 ± 6	256 ± 8

5. Absorbance Spectra

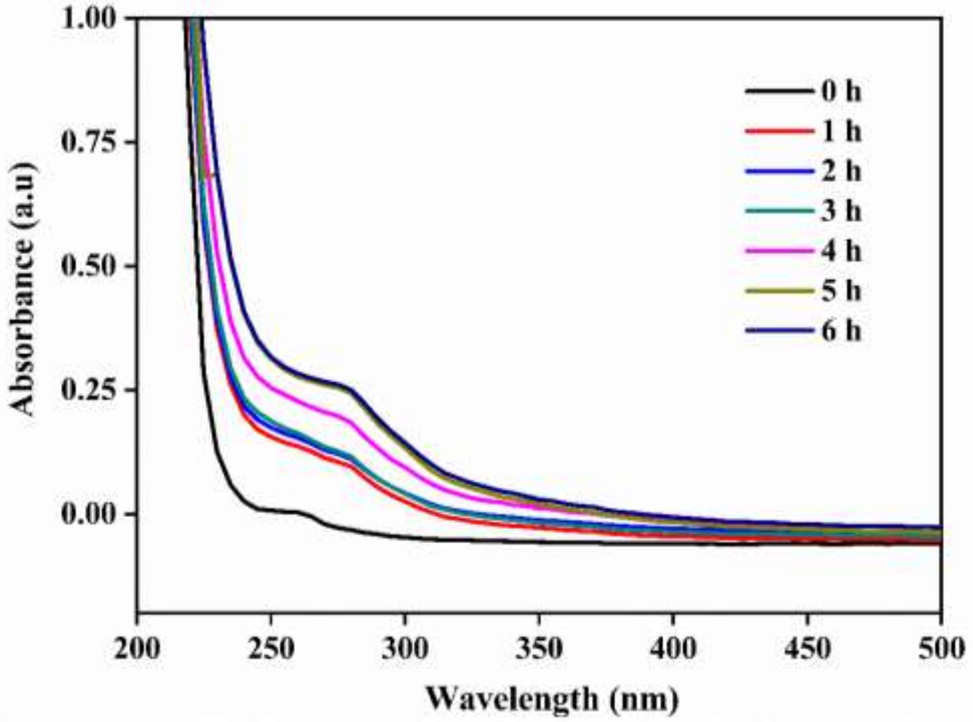

Figure S4. The absorption spectra of the $[RF]_4$ solution, acquired as a function of sonication time.

6. Size-Exclusion Chromatographic Analysis

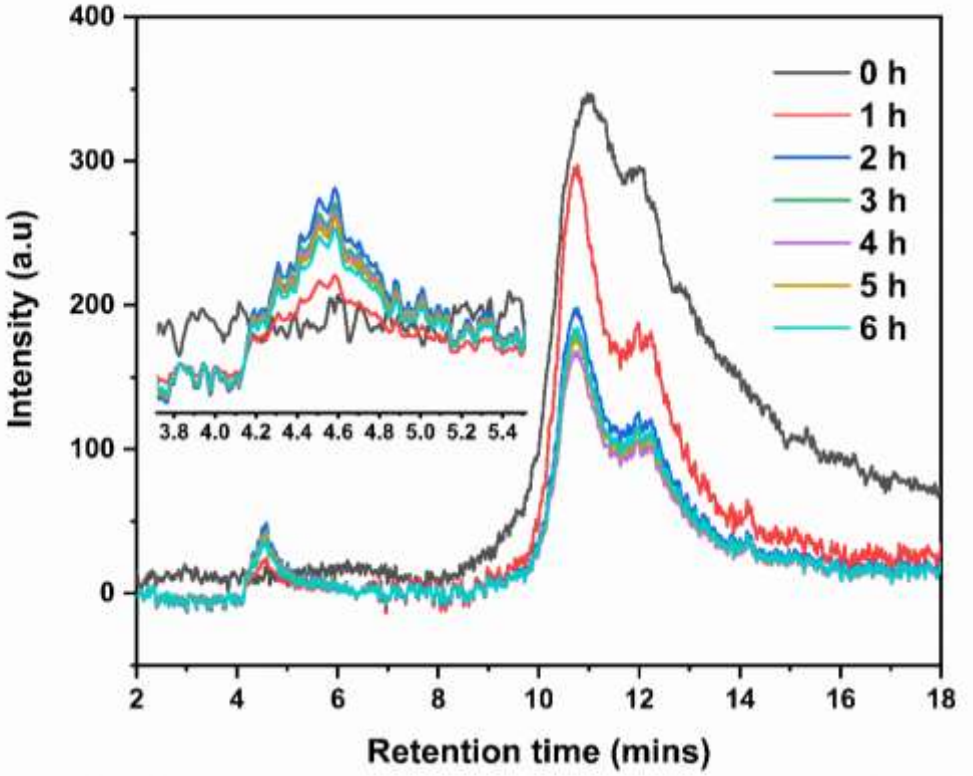

Figure S5. SEC curve of the sonicated $[RF]_4$ octapeptide as a function of sonication time.

7. ¹H NMR Analysis

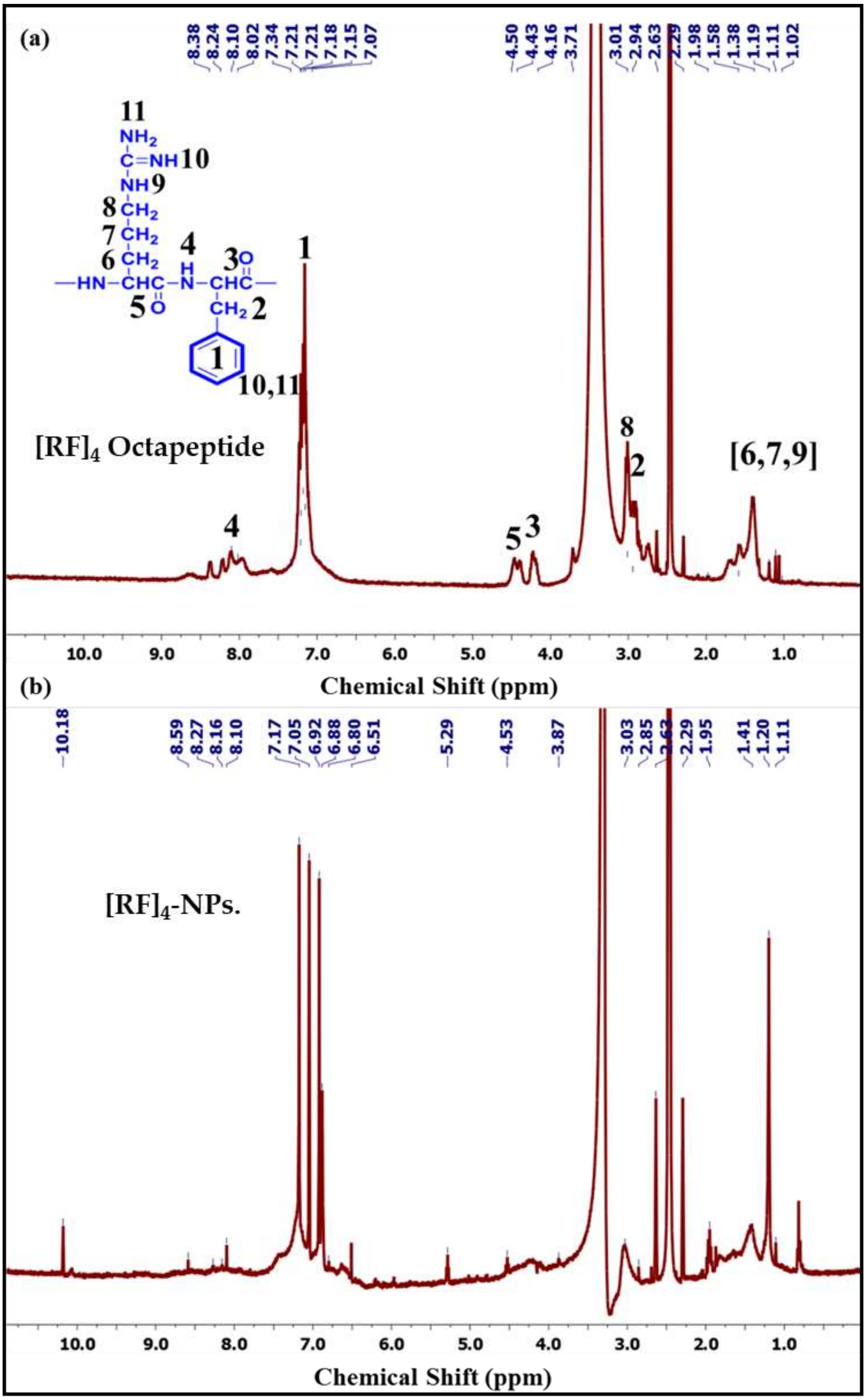

Figure S6. ¹H NMR plot of (a) $[RF]_4$ peptide, and (b) sonicated $[RF]_4$ peptide.

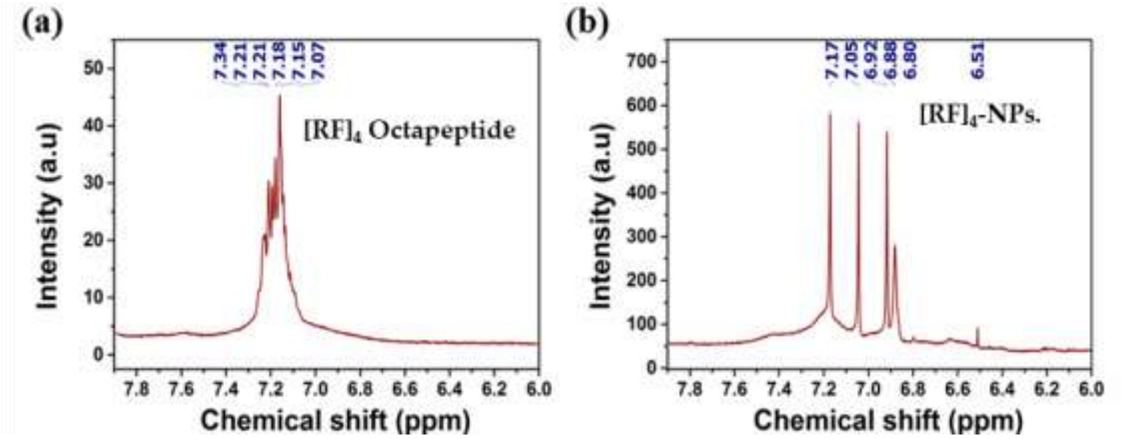

Figure S7. ^{1}H NMR chemical shifts in the aromatic region for (a) [RF]$_4$ octapeptide, and (b) [RF]$_4$-NPs.

8. PEGylated [RF]$_4$-NPs

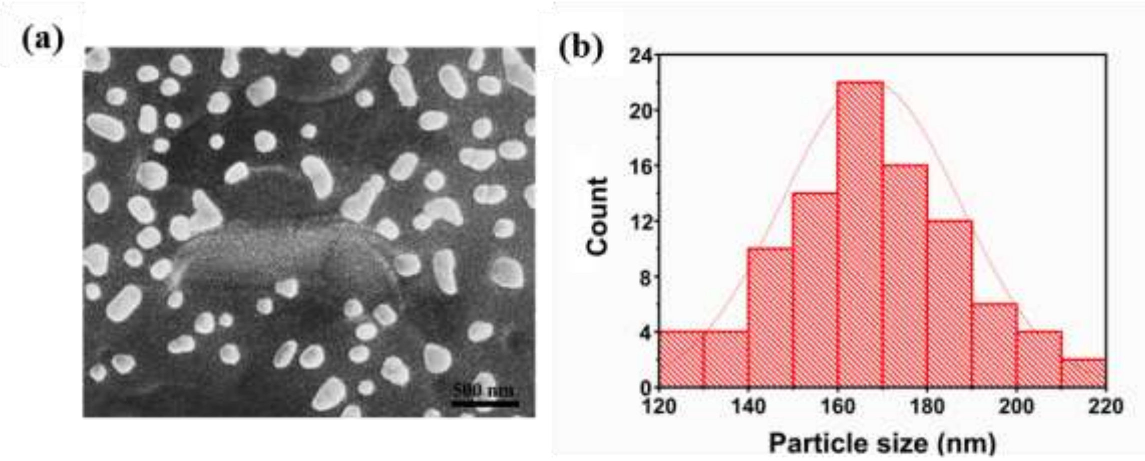

Figure S8. (a) SEM image, and (b) particle size distribution curve for the PEGylated [RF]$_4$-NPs

9. Dissolution Kinetics

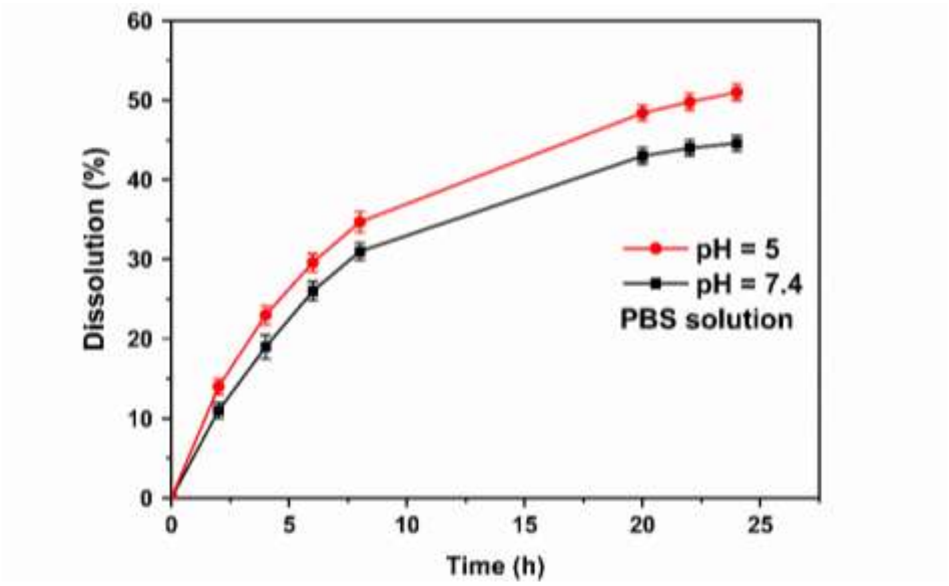

Figure S9. Dissolution kinetics of [RF]$_4$-NPs at 37 °C at different pH values as a function of time suggesting slow dissolution of [RF]$_4$-NPs at pH 5.0 and pH 7.4.

References

1. Adochitei, A.; Drochioiu, G. Rapid characterization of peptide secondary structure by ft-ir spectroscopy. *Rev. Roum. Chim* **2011**, *56*, 783-791.

© 2020 by the authors. Submitted for possible open access publication under the terms and conditions of the Creative Commons Attribution (CC BY) license (http://creativecommons.org/licenses/by/4.0/).
